# Assessing the sentinel capacity of mollusks, seawater and sediments for monitoring antimicrobial resistance in the marine environment

**DOI:** 10.3389/fvets.2025.1625423

**Published:** 2025-10-30

**Authors:** Gabriel Arriagada, Libertad Canales, Fabián Flores, Nicolás Reyes, Ismael Maldonado, Lisette Lapierre, Nicolás Galarce

**Affiliations:** ^1^Institute of Agri-food, Animal and Environmental Sciences, Universidad de O’Higgins, San Fernando, Chile; ^2^School of Agri-food, Animal and Environmental Sciences, Universidad de O’Higgins, San Fernando, Chile; ^3^Master’s Program in Biological Sciences, Faculty of Sciences, Universidad del Bío-Bío, Chillán, Chile; ^4^Department of Preventive Animal Medicine, Faculty of Veterinary and Livestock Sciences, Universidad de Chile, Santiago, Chile

**Keywords:** antimicrobial resistance, marine environment, mollusks, seawater, sediment, *Escherichia coli*

## Abstract

**Introduction:**

Although it is widely recognized that aquaculture activity is particularly relevant to the development and spread of AMR in the aquatic environment, national action plans against AMR typically do not include the marine environment among the compartments targeted for AMR monitoring and surveillance. This study aimed to compare the sentinel capacity of mollusks, seawater, and sediments for AMR surveillance in the marine environment of the Los Lagos region, Chile, using *Escherichia coli* as indicator bacteria and florfenicol, oxytetracycline, oxolinic acid, and flumequine as target antimicrobials.

**Methods:**

101 mollusk, 76 seawater and 76 sediment samples were collected simultaneously from 76 sites in the coastal area of the Los Lagos region, Chile, between 2023 and 2024. All samples were subjected to conventional laboratory procedures for *E. coli* isolation. Minimum inhibitory concentrations (MICs) for florfenicol, oxytetracycline, oxolinic acid, and flumequine were estimated for each *E. coli* isolate, which was then classified as either “wild-type” (WT; i.e., susceptible) or “non-wild-type” (NWT; i.e., tolerant) based on local epidemiological cutoff values (COwt) calculated from the MIC results. The frequency of NWT *E. coli* isolates was calculated for each of the three compartments; significant differences in the probabilities of isolating *E. coli* and detecting NWT *E. coli* were assessed using logistic regression models.

**Results:**

*E. coli* was isolated in 82.2% of the mollusk, 93.4% of the seawater, and 38.7% of the sediment samples. The COwt values were estimated in 32 μg/mL for florfenicol, 64 μg/mL for oxytetracycline, 1 μg/mL for oxolinic acid, and 2 μg/mL for flumequine. The proportion of NWT *E. coli* among the four antimicrobials was consistently higher in seawater (25.0% on average), followed by sediments (10.8%) and then mollusks (5.4%). Logistic models indicated that the probabilities of isolating *E. coli* and detecting NWT *E. coli* for the four antimicrobials studied significantly depend on the environmental compartment, with seawater having the highest probability. These results should be considered by authorities developing plans to monitor AMR in the marine environment.

## 1 Introduction

Antimicrobial resistance (AMR) is the ability of microorganisms, such as bacteria, to grow despite exposure to antimicrobial substances designed to inhibit their growth. In 2021, 1.14 million deaths were attributable to bacterial resistance, and it is estimated that by 2050, there could be 1.91 million deaths (1). The environment, particularly aquatic environments, plays a key role in the evolution and transmission of AMR as the environmental resistome constitutes a genetic reservoir of all known and unknown antimicrobial resistance mechanisms (2). Because antimicrobial concentrations in the environment are generally low compared to those found in the human-animal compartment, the development of resistance in the environment is dominated by the mobilization and transfer of antimicrobial resistance genes (ARGs) within the local bacterial community, rather than by mutations; it is thought this process is prompted by antimicrobial pollution in the environment (2).

Aquaculture farming is known for using large amounts of antibiotics, making this activity particularly relevant for selecting and spreading of AMR in aquatic environments. The role of intensive use of antibiotics in aquaculture on selection and spread in the environment of antibiotic-resistant bacteria is acknowledged by the scientific community (3). Consequently, aquaculture systems and farms are viewed as “genetic reactors” or hotspots for ARGs where significant genetic exchange and recombination can occur, which can shape the evolution of future resistance profiles (4, 5). Aquaculture-associated AMR in the environment can have two distinct origins; first, resistance may develop in the fish gut where antimicrobials come into contact with fish commensal or pathogenic bacteria; these resistant bacteria and genes eventually reach the open environment through fish feces (6, 7). Second, resistance may also develop *in situ* in environmental bacteria due to contamination by antimicrobials released into the environment through uneaten medicated feed (8, 9). As these bacteria may harbor ARGs, some embedded in mobile genetic elements, resistance can be transmitted to water and sediment bacterial communities (10). Eventually, these genes may be transferred to clinically relevant bacteria in humans (11).

Monitoring human and animal compartments is a core component of national action plans against AMR; however, the integration of the natural environment into these plans remains incomplete and unstandardized (12). Countries leading the incorporation of the environment into AMR action plans, such as Norway, are in the early stages of assessing the feasibility of implementing surveillance systems (13). However, among the different environmental niches, marine ecosystems do not appear to be a focal point of interest. Most notable advances are related to existing programs that have been extended to evaluate AMR. This is the case of the Institute of Marine Research of Norway, which annually surveys blue mussels (*Mytilus edulis*) for fecal indicator organisms such as *Escherichia coli*; since 2018, this program has also surveyed AMRin enterobacteria isolated from mussels (14, 15). Similarly, as part of their duties concerning seafood safety research and surveillance, the Norwegian Institute of Nutrition and Seafood Research conducted an initial assessment of the resistance status of enterobacteria isolated from bivalve mollusks found on the coast of the country between 2014 and 2015 (16). Recently, the UK’s Environmental Agency conducted a short study to assess the feasibility of using mollusk samples collected as part of microbiological and biotoxin monitoring programs to assess AMR. The findings of this study indicated that mollusks can be used as sentinel organisms for AMR monitoring in coastal waters (17).

Academia has also produced a substantial body of evidence that supports the use of mollusks as sentinels for monitoring AMR in the aquatic environment. Several studies have been conducted in different parts of the world to assess the resistance of various bacterial genera, including *Vibrio* [e.g., (18)], *Escherichia* [e.g., (19)], *Salmonella* [e.g., (20)], *Aeromonas* [e.g., (21)], *Enterococcus* [e.g., (22)], among others, to different classes of antimicrobials. These resistant bacteria have been isolated primarily from mussels, oysters, clams, scallops, and cockles, collected from the natural environment. However, to the best of our knowledge, no published studies have evaluated the suitability of mollusks for AMR monitoring by comparing them to other environmental compartments such as seawater and/or marine sediments, using simultaneous sampling (i.e., samples are taken from the three compartments in the same site at the same time).

The first Chilean action plan against AMR was established in 2017, and the second plan, which covers the period from 2021 to 2025, is currently in operation. The main objective of the current plan is to develop an integrated surveillance system for selected microorganisms, which will progressively include information from the human, animal, and environmental spheres (23). Currently, the plan generates information from some clinical areas and production chains, but not from the environmental compartment, including the marine environment. This situation contrasts with the significant concern in the country about the role of salmon farming in the development, maintenance, and dissemination of AMR in the marine environment (24). This industry, which has been established in southern Chile for about 30 years and covers a significant part of its coastal waters, is known to use large amounts of antimicrobials (6). According to the National Fisheries and Aquaculture Service (SERNAPESCA), the consumption of antimicrobials by the Chilean salmon industry has fluctuated greatly over the last 15 years but has shown a consistent downward trend since 2022 (25, 26). In the last decade, the most commonly used antibiotics at the seawater stage were florfenicol and oxytetracycline, together accounting for 95% of total consumption; other antibiotics used during this period were flumequine, oxolinic acid, sulfa-trimethoprim, amoxicillin, tiamulin, and tilmicosin (27–37).

The only information available on the status of AMR in the marine environment of Chile has been generated by a limited group of studies. Some of these have evaluated AMR against drugs used in salmon farming, either in marine sediments (38, 39), seawater (40), or mollusks (41). Others have assessed resistance to human and veterinary antimicrobials in wild fish (42) or in seabirds (43). None of these studies assessed resistance in more than one environmental compartment at a time, so there is no evidence to indicate whether one compartment is more appropriate than another for monitoring resistance in the marine environment.

The objective of this study was to compare the sentinel capacity of mollusks, seawater, and sediments for AMR monitoring in the marine environment of Chile. For this purpose, *E. coli* was selected as an indicator bacteria, and the antimicrobials florfenicol, oxytetracycline, oxolinic acid, and flumequine were selected as target agents.

## 2 Materials and methods

### 2.1 Study area

The study was conducted in the Los Lagos region (41°28′ to 43°36′) in southern Chile. This area accounted for about 40.86% of the country’s salmon farming activity in 2023 (26). Chiloé also has the largest shellfish production in Chile, accounting for 97% of the national output. Ninety-five percent of the production comes from mussel farms, while the remaining 5% is harvested directly from natural beds (26). Although not included in the official records, the collection of shellfish from the intertidal zone along the coast is also an important economic activity in Los Lagos. The main human settlements in this area are Puerto Montt, Calbuco, and Ancud in the north, Castro in the center, and Quellón in the south, with a total population of 850,000 inhabitants.

### 2.2 Study design

The design was a cross-sectional study, and it aimed to compare the frequency of resistant *E. coli* isolates between three environmental compartments, namely mollusks, seawater, and sediments. For this purpose, 76 sampling sites were established along the northeastern coast of Chiloé Island, Calbuco, Reloncaví Bay, Reloncaví Estuary, and Hornopirén, representing different levels of human activity and industrial influence, including salmon farming. The final location of the sampling sites was determined by their accessibility, the presence of mollusks in the intertidal zone, and/or the existence of a shellfish farm from which mollusks could be obtained. One or more mollusk samples, one seawater sample, and one sediment sample were collected at each sampling site. Samples were collected in four campaigns distributed over June 2023 and April 2024.

### 2.3 Sample collection

Mollusk samples were collected from the intertidal zone at times of low tide using a shovel. Each sample consisted of a group of individuals of the same mollusk species in sufficient numbers to collect 100 g of soft tissue. If more than one species of mollusk was found at a particular sampling site, one sample was collected for each of the species, resulting in a sampling site with multiple mollusk samples. On a few occasions, mollusk samples were collected directly from shellfish farms. Mollusk samples were individually placed in plastic bags labeled with the sampling site code and the species. Water samples of 1 L were collected directly from the sea in 1.5 L plastic bottles previously washed with potable water. When the mollusk sample was collected from a shellfish farm, the water sample was collected from the farm’s surrounding waters. Sediment samples of 50 mL were collected from the intertidal zone using individual Falcon tubes; when the mollusk sample was obtained from a shellfish farm, the sediment sample was collected from the shore in front of the farm. The geolocation of each sampling site was recorded under the WGS84 datum using a Garmin GPS, model GPSMPA 64sc. All samples were individually labeled with the sampling site code and stored at refrigerated temperature until laboratory processing, which occurred within 24 h. All samples were treated and analyzed individually, even for samples obtained from the same sampling site.

### 2.4 Laboratory procedures

#### 2.4.1 Sample processing

Once in the laboratory, mollusks from each sample were shelled using a sanitized shucking knife to obtain 100 g of soft tissue and intravalvar liquid. The contents were placed in sterile filter bags (Bagfilter®) with 100 mL of sterile 0.1% peptone diluent (1:1) and transferred to a homogenizer (Stomacher® 400 Circulator, Seward Ltd.) for 15 s. Each seawater sample was filtered using a vacuum filtration pump with a 0.45 μm-pore nitrocellulose membrane to recover *E. coli*. Membranes were prepared for inoculation onto tryptone bile X-glucuronide (TBX) agar plates (44). For sediment samples, two grams of sediment were extracted and then 18 mL of buffered peptone water were added to achieve a 1:10 concentration. The tubes were then shaken in an incubator shaker at 150 rpm for 30 min. The supernatant was then filtered using a vacuum filtration pump and a sterile membrane filter (0.45 μm) (45). Next, the filter was prepared for inoculation onto TBX agar plates.

#### 2.4.2 *E. coli* isolation and enumeration

The most probable number (MPN) method, according to Walker et al. (46), was performed to estimate the concentration of viable *E. coli* in mollusk samples. To that end, three series of five tubes were filled with MMGB containing 1, 0.1, and 0.01 g of sample, respectively. The tubes were then incubated at 37 ± 1 °C for 24 ± 2 h, after which each tube that had turned yellow was spread onto TBX agar plates. The plates were then incubated at 44 °C for 21 ± 3 h. Positive results for the presence of *E. coli* were identified as greenish-blue colonies (44). Colonies were selected and stored at −20 °C in cryotubes for subsequent analysis. The number of plates with greenish-blue colonies from each dilution provided the MPN, which was then compared with the respective table to determine the number of colony-forming units in CFU per 100 g. For seawater and sediment samples, the membranes placed in the TBX plates were incubated at 44 °C for 21 ± 3 h. The presence of greenish-blue colonies was considered indicative of a positive sample. Enumeration was performed by directly counting the greenish-blue colonies on each membrane using a Suntex colony counter model 570, and expressing the result as CFU/100 g. Colonies were then selected by extracting them with a loop and stored at −20 °C in cryotubes for subsequent analysis.

#### 2.4.3 Antimicrobial susceptibility testing

Phenotypic resistance was assessed by estimating the minimum inhibitory concentration (MIC). MIC was estimated using the plate microdilution method (47). For this, 96-well U-bottom plates were previously filled with the antibiotics florfenicol, oxytetracycline, oxolinic acid, and flumequine. Twelve concentrations ranging from 0.25 to 512 μg/mL were used, with 50 μL of the drug filled into each well. Samples were analyzed in duplicate, and strain *E. coli* ATCC 25922 was used for quality control, as recommended by the Clinical and Laboratory Standards Institute (CLSI) (47). To prepare the inoculum, previously frozen beds in cryo-tubes were extracted and spread on Tryptic Soy Agar (TSA). The plates were then incubated for 24 h at 35 °C to allow the growth of *E. coli* colonies. Then, the suspension was transferred to Mueller-Hinton 2 (MH2), and its optical density was measured using a photometric device to obtain a suspension approximately 1 to 2 × 10^8^ CFU/mL. Finally, 50 μL of the inoculum were placed in each well of the microplate, resulting in a final volume of 100 μL (5 × 10^5^ CFU/mL). The plates were then incubated at 35 ± 2 °C for 16 to 20 h. MICs were determined by the unaided eye.

### 2.5 Statistical procedures

#### 2.5.1 Categorization of isolates as wild-type or non-wild-type

*E. coli* isolates from each compartment were categorized as wild-type (WT) or non-wild-type (NWT), based on the establishment of a local epidemiologic cut-off value (COwt), which was calculated from the observed MIC values. WT isolates were those that did not show any phenotypic resistance in antimicrobial susceptibility testing, whereas NWT isolates showed variable levels of tolerance (47). COwt represents the upper MIC limit of the distribution obtained from the fully susceptible members (i.e., WT population) of a bacterial species. Antimicrobial-specific COwt was calculated using the Normalized Resistance Interpretation (NRI) method (48), implemented in an automated spreadsheet available at https://www.bioscand.se/nri/. Briefly, the NRI method reconstructs the WT population in a MIC distribution for a given bacterial species challenged with a particular antimicrobial agent, producing a normal distribution for WT isolates; subsequently, cut-off values are set at +2.0 SD above the mean of the reconstructed normal distribution. In our case, the COwt was calculated considering the MIC values of all isolates, regardless of the type of sample from which they originated; therefore, the same COwt was used to categorise isolates in each of the three compartments. This procedure was repeated for each of the four antimicrobials studied.

#### 2.5.2 Comparing the sentinel capacity of the mollusk, seawater, and sediment compartments for AMR monitoring in the marine environment

The suitability of mollusks for monitoring resistant *E. coli* compared to seawater and sediment was assessed using two logistic regression models. The first model determined which compartment had a higher probability of *E. coli* isolation, regardless of WT or NWT status. The second model estimated whether NWT *E. coli* were more likely to be isolated from a particular compartment. The outcome of the first model was the log-odds of isolating *E. coli*, while the outcome of the second model was the log-odds of isolating an NWT *E. coli* strain. The main exposure in both models was the compartment from which the sample was collected, represented by a 3-level categorical variable, with the categories being mollusks (reference), seawater, and sediment. Because contexts with a high bacterial load are prone to the development and transmission of AMR (49), a four-level categorical variable representing the bacterial load was included in the second regression model. Categories were defined as low (reference), medium, high, and very high bacterial load. This variable was constructed from the results of *E. coli* enumeration measured in each sample, either MPN/100 g in the case of mollusk samples, or CFU/L or CFU/g, in the case of water and sediment samples, respectively, by establishing cut-off points at the 25th, 50th, and 75th percentiles. The second model was built for each of the four antimicrobials under study. The equations for the two models are as follows:


(1) $$\text{logit}(P)=\beta_{0}+\beta_{1}(\text{seawater})+\beta_{2}(\text{sediments})$$

(2) $$\begin{array}{l} \text{logit}(P)=\beta_{0}+\beta_{1}(\text{seawater})+\beta_{2}(\text{sediments})+\beta_{3}(\text{medium})+\\ \beta_{4}(\text{high})+\beta_{5}(\text{very high})\; \end{array}$$

where *P* is the probability of isolating *E. coli* (Equation 1) or the probability of isolating a NWT *E. coli* strain (Equation 2); $\beta_{0}$ is the constant; $\beta_{1,2}$ are the regression coefficients associated with the environmental compartment, and $\beta_{3-5}$ are the regression coefficients associated with the bacterial load.

In the two models, the strength of associations between predictors and the outcome was expressed as odds ratios (OR). Model fit will be assessed using the Pearson $\chi^{2}$ statistic, as recommended when the model includes categorical variables (50).

Patterns of isolation and resistance at the site level were explored using multiple correspondence analysis (MCA). Specifically, MCA evaluated the proximity of three possible conditions per antimicrobial—no isolation, WT, or NWT—across sites. For sites with multiple mollusk samples, the sample with the worst condition (WT or NWT) was used to represent the site. MCA was limited to the derivation of two dimensions. The relationships between the isolation and resistance conditions between sampling sites were plotted by a two-dimensional correspondence map. All statistical analyses were performed using Stata IC version 15 (StataCorp, College Station, TX, United States).

## 3 Results

A total of 101 mollusk samples were collected, of which 87.1% belonged to the class Bivalvia and 12.9% to the class Gastropoda. The most collected bivalve species were mussels (81.2%), including *Mytilus chilensis*, *Perumytilus purpuratus*, *Aulacomya atra*, and *Choromytilus chorus*, clams (4.0%), and oysters (2.0%), while gastropods were mainly represented by limpets (*Fissurella* sp.; 7.9%) and snails (*Tegula atra*; 4.9%). A single mollusk sample was collected at 57 sampling sites, two samples were collected at 16 sites, and three, four, or five samples were collected at one site. As planned, 76 seawater samples and an equal number of sediment samples were collected from the sampling sites.

*E. coli* was isolated from 82.2% of the mollusk samples, 93.4% of the water samples, and 38.7% of the sediment samples. Among mollusks, *E. coli* was found in 83.0% of bivalves and 76.9% of gastropods. After adjusting for multiple comparisons, model 1 shows that the probability of isolating *E. coli* was significantly higher in mollusks (*p* < 0.001) and seawater (*p* < 0.001) when compared to sediments. However, there were no significant differences between mollusks and seawater samples (*p* = 0.102).

MIC median values for florfenicol in the three compartments were 16 μg/mL with a minimum ranging between 4 and 8 μg/mL, and a maximum of ≥512 μg/mL. In the case of oxytetracycline, the median of MIC was 8 μg/mL (range 1 – ≥ 512 μg/mL) in mollusks, 16 μg/mL (range 1– ≥ 512 μg/mL) in seawater, and 8 μg/mL (range 2– ≥ 512 μg/mL) in sediments. MIC median values for oxolinic acid reached 0.25 μg/mL (range ≤0.25–128) in mollusk samples, 0.5 μg/mL (range ≤0.25– ≥ 512 μg/mL) in seawater samples, and 0.25 μg/mL (range ≤0.25– ≥ 512 μg/mL) in sediment samples. Finally, median MIC values for flumequine across compartments were 0.50 μg/mL (range ≤0.25–128 μg/mL) in mollusks, 1 μg/mL (range 0.5–≥ 512 μg/mL) in seawater, and 0.50 μg/mL (range ≤0.25– ≥ 512 μg/mL) in sediments (Table 1).

**TABLE 1:** Distribution of minimum inhibitory concentrations (MIC) for florfenicol, oxytetracycline, oxolinic acid, and flumequine for the 184 *E. coli* strains isolated from the mollusk, seawater, and sediment compartments.

Anti-microbial	Compartment	Minimum inhibitory concentration (μg/mL)												p50	n	% NWT*
		≤0.25	0.5	1	2	4	8	16	32	64	128	256	≥512			
Florfenicol	Mollusks					4	18	51	8				2	16	83	2.41
	Seawater						18	29	12	2		3	7	16	71	16.90
	Sediments						11	15	2	1			1	16	30	6.67
Oxy-tetracycline	Mollusks			4	3	5	34	23	1	2		8	3	8	83	13.25
	Seawater			2	2	7	22	10	4	4	1	11	8	16	71	28.17
	Sediments				2	8	12	3	1			2	2	8	30	13.33
Oxolinic acid	Mollusks	59	16	6			1				1			0.25	83	2.41
	Seawater	22	22	8	2	8	1	3	1				4	0.50	71	26.76
	Sediments	18	7	1	1		1			1			1	0.25	30	13.33
Flumequine	Mollusks	5	61	12	2	1			1		1			0.50	83	3.61
	Seawater		25	23	3	4	3	1	2	3	3	1	3	1	71	28.17
	Sediments	4	18	3	2			1			1		1	0.50	30	10.00

Cells in gray depict *E. coli* strains that had MIC values above the antibiotic-specific COwt value. *NWT, non-wild-type isolates.

COwt values were calculated as 32 μg/mL for florfenicol, 64 μg/mL for oxytetracycline, 1 μg/mL for oxolinic acid, and 2 μg/mL for flumequine. Accordingly, the percentages of NWT *E. coli* strains for florfenicol were 2.4% in mollusks, 16.9% in seawater, and 6.7% in sediments. In the case of oxytetracycline, NWT *E. coli* isolates accounted for 13.3% in mollusks, 28.2% in seawater, and 13.3% in sediments. The percentages of NWT isolates of *E. coli* for oxolinic acid across compartments were 2.4, 26.8 and 13.3% for mollusks, seawater and sediments, respectively. Finally, in the case of flumequine *E. coli* NWT strains represented 3.6% in mollusk, 28.2% in seawater and 10.0% in sediment samples (Table 1). Among the mollusk samples from which NWT *E. coli* could be isolated, 89% were bivalves.

The median concentration of *E. coli* in mollusk samples was 170 MPN/100 g with a range of 20 to 9,200 MPN/100 g. In seawater samples, the median *E. coli* load was 26 CFU/L with a range between 1 and 500 CFU/L, while in sediment samples, the median *E. coli* concentration was 3 UFC/g with a range between 1 and 56 CFU/g.

Model 2 shows that the probability of isolating NWT *E. coli* strains significatively depends on the environmental compartment for the four antimicrobials under study; in particular, this dependency was highly dominated by significant differences observed between the seawater and the mollusk compartments. After penalizing for multiple comparisons NWT *E. coli* strains were significantly more likely to be isolated from seawater samples than from mollusk samples when testing for florfenicol (OR = 8.2, *p* = 0.021), oxolinic acid (OR = 20.0, *p* = 0.001), and flumequine (OR = 12.9, *p* < 0.001; Tables 2, 3). It was also more likely to isolate NWT *E. coli* from seawater than from mollusks when testing for oxytetracycline, but this association was borderline significant (OR = 20.0, *p* = 0.001). No significant differences were found between mollusks and sediments, or between seawater and sediments for the four antimicrobials (Tables 2, 3). Model 2 also indicates that variations in *E. coli* load in samples significantly impacted the probability of isolation of NWT *E. coli* strains, but only when monitoring resistance to oxytetracycline (*p* = 0.001), oxolinic acid (*p* = 0.001), or flumequine (*p* = 0.004), especially when comparing low to very high *E. coli* loads (Table 2).

**Table 2:** Logistic regression models comparing the likelihood of detecting NWT *E. coli* in the mollusk, seawater, and sediment compartments for the four antimicrobials studied.

Variable	Florfenicol			Oxytetracycline			Oxolinic acid			Flumequine		
	Odds ratio	Standard error	*p*-value*	Odds ratio	Standard error	*p*-value*	Odds ratio	Standard error	*p*-value*	Odds ratio	Standard error	*p*-value*
Intercept	0.025	0.018	**<0.001**	0.105	0.061	**<0.001**	0.017	0.015	**<0.001**	0.033	0.026	**<0.001**
Compartment (mollusk as reference)			**0.018**			**0.043**			**0.001**			**<0.001**
Seawater	8.237	6.447	**0.007**	2.866	1.273	**0.018**	20.039	16.056	**<0.001**	12.917	8.697	**<0.001**
Sediment	2.893	2.962	0.299	1.098	0.732	0.889	8.047	7.594	**0.027**	3.636	3.224	0.145
*E. coli* load (low as reference)						**0.001**			**0.001**			**0.004**
Medium				0.315	0.247	0.141	0.239	0.225	0.128	0.194	0.176	0.070
High				1.345	0.847	0.638	0.637	0.507	0.571	0.658	0.478	0.564
Very high				3.781	2.257	**0.026**	4.359	3.078	**0.037**	2.953	1.943	0.100

*Bolded *p*-values indicate significant variables (*p* ≤ 0.05).

**Table 3:** Pairwise comparisons of marginal linear predictions of the log odds of NWT *E. coli* detection across environmental compartments, based on models shown in Table 2.

Comparison	Florfenicol		Oxytetracycline		Oxolinic acid		Flumequine	
	Contrast	*p* value	Contrast	*p* value	Contrast	*p* value	Contrast	*p* value
Seawater vs. mollusks	2.109	0.021	1.053	0.053	2.998	0.001	2.556	<0.001
Sediment vs. mollusks	1.062	0.898	0.093	1.000	2.085	0.081	1.291	0.436
Sediment vs. seawater	−1.046	0.797	−0.960	0.414	−0.912	0.554	−1.268	0.245

*p-*values are adjusted with the Bonferroni method.

**Figure 1:**
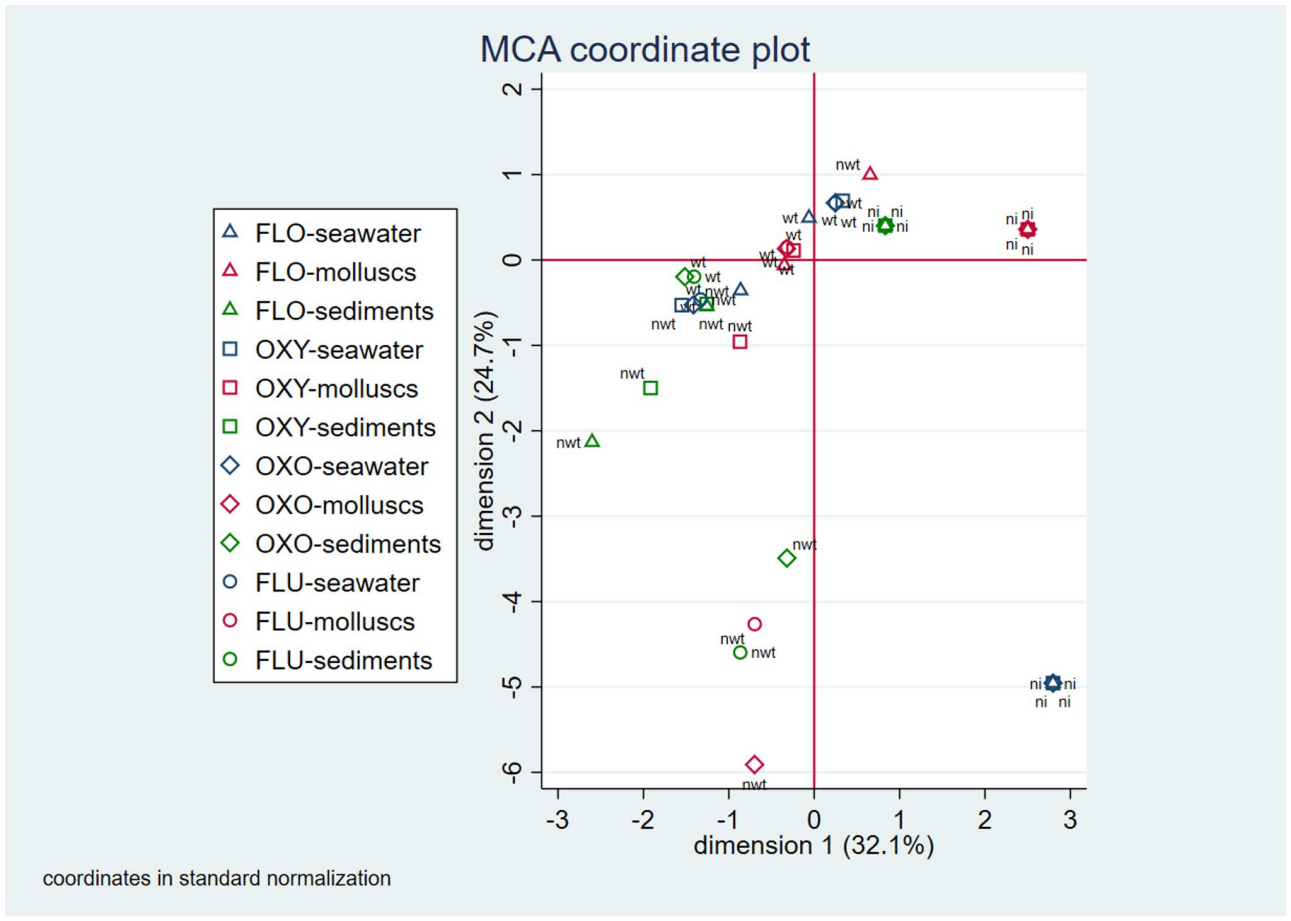
Two-dimension correspondence map for patterns of *E. coli* isolation and NWT *E. coli* detection observed at the sampling site level (ni, no isolation; wt, wild-type; nwt: non-wild-type; FLO, florfenicol; OXY, oxytetracycline; OXO, oxolinic acid; FLU, flumequine).

The first and the second derived dimension in the MCA analysis together accounted for 56.8% of the total variability of the isolation and resistance status across sampling sites (first dimension = 32.1%; second dimension = 24.7%). The two-dimensional correspondence plot shows different groups of conditions that correspond to identifiable isolation-resistance patterns. When WT *E. coli* were isolated from seawater, they were usually also isolated from mollusks, but not from sediments, where the frequency of *E. coli* isolation was lower. In contrast, when oxytetracycline, oxolinic acid, or flumequine NWT *E. coli* was detected in seawater, they were usually not found in either mollusks or sediments. In cases when the NWT *E. coli* detected in seawater was associated to florfenicol, it was never detected in mollusks; however, NWT bacteria related to the other three antimicrobials were generally detected in the same or different compartments (Figure 1). The most commonly found resistance profiles were “oxytetracycline-oxolinic acid-flumequine” (26%), predominant in seawater, and “oxytetracycline” (26%), predominant in mollusks. Other notable resistance profiles were “florfenicol” (12%), which was only present in *E. coli* isolated from seawater; “florfenicol-oxytetracycline-oxolinic acid-flumequine” (10%), which was only present in seawater and sediments; “oxolinic acid-flumequine” (10%), which was predominant in seawater; and “florfenicol-oxytetracycline” (8%), which was present in all three compartments. Profiles with frequencies lower than 5% were “flumequine,” “oxolinic acid,” and “florfenicol-oxolinic acid-flumequine” (Table 4).

**Table 4:** Resistance profiles of NWT *E. coli* isolates by environmental compartment.

Resistance profile	Compartment			Frequency
	Mollusks	Seawater	Sediments	
OXY-OXO-FLU	1	11	1	13
OXY	8	4	4	13
FLO	0	6	0	6
FLO-OXY-OXO-FLU	0	4	1	5
OXO-FLU	1	3	1	5
FLO-OXY	2	1	1	4
FLU	1	1	0	2
FLO-OXO-FLU	0	1	0	1
OXO	0	0	1	1
Total	13	31	6	50

FLO, florfenicol; OXY, oxytetracycline; OXO, oxolinic acid; FLU, flumequine.

## 4 Discussion

This study aimed to evaluate the convenience of using mollusks for AMR monitoring in the marine environment compared to both the seawater and the sediment compartments. As a first aspect to consider, it is important to highlight that the compartment with the highest probability of detecting *E. coli* was seawater, contradicting the idea that mollusks, due to their ability to concentrate pollutants in their organism (51), will generally have a greater bacterial load than other environmental compartments. In fact, our results indicate that in some sampling sites, *E. coli* was detected in seawater only; this could be the result of pulses of microbiological contamination that first impact the seawater and then spread to other compartments in the marine environment. Since the level of fecal coliform in seawater is affected by environmental variables (e.g., salinity, temperature, solar radiation, and rainfall) (52) and the proximity to pollution sources (53), the probability of isolating *E. coli* from seawater, sediments or microbiota is dependent on local-scale factors. This is consistent with a notable variability in the results of studies aimed at quantifying or detecting (fecal) coliforms or *E. coli* in different compartments of aquatic environments; in some of these studies it was reported that bacteria contamination was more frequently detected in mollusks or sediments than in seawater [e.g., (54)], in others the percentage of isolation in seawater was higher than in the mollusk [e.g., (55)], while in others mixed results were reported [e.g., (56)]. Our results may be explained by the high spatio-temporal variability of the fecal coliform load in seawater in the study area, characterized by relatively low mean values in most of the locations (e.g., Calbuco: 3.0 MPN/100 g, Canal Dalcahue: 3.0 MPN/100 g, Canal Yal: 3.0 MPN/100 g, Pargua: 1.0 MPN/100 g, Quellón: 8.0 MPN/100 g), but with significant temporal peaks in areas near Puerto Montt (mean: 1,121 MPN/100 g) and Castro (mean: 297 MPN/100 g) (57). Local conditions in the study area may also explain the relatively greater proportion of *E. coli* isolation from mollusks in our study (82.2%), compared to similar studies in Norway, where the proportion of bacterial isolation in mollusks was 67% for *E. coli* (15) and 36% for enterobacteriaceae, including *E. coli*, *Klebsiella*, *Citrobacter* and *Enterobacter* (16). In any case, our results should be interpreted in light of the fact that we did not perform a specific confirmatory test during the isolation and enumeration of *E. coli*; rather, we relied on the selective capacity of the TBX growth medium. This may result in the growth of bacteria other than *E. coli* with some *β*-glucuronidase capacity, such as *Shigella*, *Klebsiella*, *Enterobacter*, or *Citrobacter* (58). However, a study using TBX agar as a selective medium and MALDI-TOF as a confirmatory method for water samples determined that the probability of false positives was only 0.7% (44). Therefore, while we acknowledge the possibility of having isolated bacteria other than *E. coli* in our study, this possibility is very low.

Although the seawater compartment appears to be the most suitable for isolating *E. coli*, the characteristics of water, particularly when affected by currents and wind, imply that contaminants exhibit a very high spatial and temporal variability. This can result in inconsistent results when samples are taken from the same location at different times, which is an undesirable feature for a monitoring system. Sediments, however, reflect a more stable reality over time as they provide favorable conditions for the survival of fecal indicator bacteria (59), thus ensuring more consistent results. The relatively low percentage of *E. coli* isolation in sediments in our study may be due to the lower bacterial retention capacity described in sandy beaches due to their larger sediment grain size in the intertidal zone (60), which was the type of beaches from which we collected our samples.

Failing to incorporate environmental variables in this study may significantly limit the interpretation of differences observed between the three environmental compartments. Temperature and salinity have been identified as environmental factors that significantly impact the level of fecal coliforms in the marine environment (52). Similarly, an increase in seawater temperature has been linked to a higher frequency of horizontal gene transfer and to a heat shock response in *E. coli* (61). Furthermore, salinity has been reported to significantly affect the composition of resistance genes in marine bacteria (62). Therefore, as temperature, salinity and other environmental variables were not considered, these findings should be interpreted with caution.

Since mineral water bottles were used to collect seawater samples instead of for sterile, quality assurance (QA)-verified sampling containers, the possibility of sample contamination cannot be ruled out. However, given Chile’s strict regulations governing drinking and bottled water, which require the absence of *E. coli* per 100 mL (0 CFU/100 mL) (63), the likelihood of sealed water bottles being contaminated with this bacterium and reaching retail outlets is extremely low.

In general, the observed range of MIC values for the same antibiotic in mollusks, seawater and sediments were similar, suggesting that resistant bacteria populations and ARGs move across the different environmental compartments, and/or that the concentration of antimicrobials in the three compartments is high enough to exert equivalent ecotoxicological effects. Exceptions to this pattern were observed for oxolinic acid and flumequine, where the highest MIC values observed in mollusks were two concentrations lower than those recorded in seawater and sediments (128 vs. 512 μg/mL). This might be explained by the low bioaccumulation in benthic macrobiota described in general for quinolones (64), and in particular for flumequine and oxolinic acid when compared to oxytetracycline (65, 66). This is consistent with the lower recovery percentage of flumequine with respect to oxytetracycline and florfenicol found in marine invertebrates in the context of recovery studies (67). It is important to note that the lowest MIC values for florfenicol and oxytetracycline in mollusks observed in our study were substantially higher than those reported by Ramírez et al. (41) for the same antimicrobials in heterotrophic bacteria isolated from mussels (*Mytilus* spp.) in southern Chile. Moreover, the highest MIC values for oxytetracycline in mollusk-isolated *E. coli* described in our study were two orders of magnitude higher than those reported for the bacterial community studied by Ramírez et al. (41) (128 vs. ≥512 μg/mL). These facts suggest that the *E. coli* isolated from mollusks in our study represent a bacterial population that was somehow more exposed to these antimicrobials than typical shellfish microbiota from the same geographical area, which are comparatively more sensitive.

Our findings indicate that the seawater compartment contained a higher frequency of MIC values above the COwt (i.e., % NWT isolates) compared to mollusks and sediment samples; this occurred for the four antimicrobials studied. In the case of florfenicol, the percentage of NWT *E. coli* isolates in seawater was seven times higher than in mollusks and 2.5 times higher than in sediment samples. Considering that a key factor in the development of bacterial resistance in the marine environment is the concentration of antimicrobials in the different compartments (2), these results are expected since florfenicol is a molecule with a low tendency to associate with particles, thus remaining in the seawater longer than in other compartments of the marine environment (68); this is confirmed by experiments where florfenicol is no longer detectable in sediments and benthic macrofauna as early as 1 week after application (69) and by observational studies where florfenicol is also not detected in sediments at a site only 20 meters away from an active farm (38). The relatively low proportion of florfenicol-NWT *E. coli* found in mollusks in our study (2.4%) is consistent with studies conducted in Norway, where the frequency of amphenicol-resistant enterobacteriaceae isolated from mussels was 5% (16). This suggests that despite the sustained use of florfenicol by the respective salmon industries (25, 70), mussels do not appear to be significant reservoirs of resistance associated with this antimicrobial.

Considering that between 2017 and 2021 the odds of florfenicol to oxytetracycline treatments in the Chilean salmon industry was 2.6:1 (71), at first glance, it seems paradoxical that the percentage of NWT *E. coli* for oxytetracycline in the three compartments in our study was substantially higher than that for florfenicol. The greater level of resistance to oxytetracycline, despite its relatively lower use, may be in part explained by the higher persistence of oxytetracycline in marine sediments and benthic macroinvertebrates compared to florfenicol, as demonstrated by a field experiment conducted by González-Gaya et al. (69). Another study carried out between 2018 and 2019 in a few locations within the same area of our study reported a higher percentage of resistance to florfenicol than to oxytetracycline (19.2% vs. 8.4%) in bacteria isolated from mussels (*Mytilus* spp.) (41). A closer look at the supplementary data of this study revealed that florfenicol was used 370 times more than oxytetracycline in terms of defined daily doses (DDDvet) in the salmon farm neighborhoods where the mussel sampling sites were located, suggesting that local AMR is strongly influenced by the particular conditions of antimicrobial use in nearby salmon farms. Regarding the high percentage of *E. coli* NWT in seawater observed in our study, it is interesting to note that another study carried out in the same area obtained comparable results. In this study, 28.3% of the bacterial strains isolated from seawater samples were resistant to oxytetracycline (40); however, it is important to note that the samples were obtained from only two sites, so their representativeness for the study area may be questioned. The relatively high proportion of oxytetracycline-resistant *E. coli* strains in the seawater compartment may be due to the fact that this agent has a high solubility in water (72), which determines that when used in aquaculture, it is present in relatively high concentrations in the water phase compared to sediments (72, 73). The proportion of oxytetracycline NWT *E. coli* isolated from mollusks in our study was 13.25%, which is greater than the reported prevalence of tetracycline-resistant enterobacteriaceae (8%) and *E. coli* (5.7%) isolated from mollusks in Norway (15, 16).

The presence of NWT *E. coli* isolates for the quinolones oxolinic acid and flumequine was surprising given that these active ingredients have not been used in salmon farming in Chile since 2014 (29) and 2018 (33), respectively. However, studies evaluating the impact of antimicrobial-free animal production systems have shown that the decline of phenotypic resistance following the cessation of antimicrobial use (i.e., phenotypic reversion) can take several years, depending on the bacterial species and the antimicrobial resistance mechanism (74). Interestingly, a meta-analysis found that among several antimicrobials evaluated (fluoro)quinolones was the only antimicrobial class associated with a higher prevalence of resistant bacteria in animal farms without antimicrobial use, highlighting past (fluoro)quinolone use as a potential determinant (75). This may indicate that resistance to quinolones is more persistent than to other antimicrobials in the environment. It is important to note that the proportion of quinolone-NWT *E. coli* observed in mollusks in our study (2.4% for oxolinic acid and 3.6% for flumequine) was similar to that found in enterobacteriaceae and *E. coli* isolated from mussels in Norway (15, 16).

If it is assumed that the observed differences in the proportion of NWT *E. coli* across environmental compartments is a reflect of the differential accumulation of antimicrobials in these compartments, the relatively low proportion of NWT *E. coli* isolates for oxolinic acid in mollusks and sediments compared to seawater may be explained by its relatively low likelihood to be transferred from seawater to sediments (76), low bioaccumulation rate in aquatic organisms (66) and a relatively fast natural attenuation in marine sediments (half-life of 26.7 days) (76). Following the same argumentation, the greater proportion of NWT *E. coli* for flumequine in seawater may be explained by its great affinity for suspended particles compared to sediments, which explains that flumequine has been detected in seawater nearby salmon farms but not in their sediments 12 months after a flumequine treatment (77).

It is important to note that among all the pairwise comparisons of the percentage of NWT isolates between compartments, only the comparison between seawater and mollusks was statistically significant, favoring the seawater compartment (Table 3). These significant differences were observed when evaluating florfenicol, oxolinic acid, and flumequine; differences favoring the mollusk compartment were also observed in the case of oxytetracycline, but they were borderline significant (Table 3). Our results also suggest that the probability of detecting NWT *E. coli* isolates increases as the concentration of *E. coli* in the compartment increases; this is consistent with previous research that has identified fecal pollution as a key driver of antimicrobial resistance in anthropogenically impacted environments (49, 78), and particularly in aquaculture settings where an increased risk of detecting resistant *E. coli* was found for samples with *E. coli* concentrations above the threshold for direct human consumption (15). The persistence of quinolone resistance in seawater, mollusks, and sediments demonstrates that aquaculture activity may have long-term effects on the marine environment. This suggests that environmental AMR surveillance programs should monitor resistance to both currently and previously used antimicrobials. This is particularly important for the Chilean salmon industry, given that a variety of antibiotics have been used sporadically or for short periods in the past, in addition to the widely used florfenicol and oxytetracycline. It is not yet known whether environmental bacteria have developed resistance to these antimicrobials.

From a practical point of view, the likelihood of detecting NWT *E. coli* strains in a particular environmental compartment is given by the probability of isolating *E. coli* in the compartment multiplied by the probability of the isolated *E. coli* strain being NWT in that compartment. For example, according to models 1 and 2, the probability of isolating *E. coli* from seawater and the probability of that *E. coli* being NWT for florfenicol in the same compartment are 0.934 and 0.169, respectively, resulting in a joint probability of 0.158. Following the same procedure, the calculated joint probability of detecting florfenicol NWT *E. coli* strains for mollusks was 0.020, while for the sediment compartment it was 0.026. This suggests that mollusks are the sample type least likely to successfully detect florfenicol-NWT *E. coli* strains. Again, according to models 1 and 2, the joint probability of detecting NWT *E. coli* strains in the seawater compartment for oxytetracycline, oxolinic acid, and flumequine was 0.260, 0.245, and 0.256, respectively, which exceeded that of the mollusk compartment by between 2 and 12 orders of magnitude, and that of the sediment compartment by between 4 and 8 orders of magnitude. According to this rationale, the more appropriate compartment for monitoring AMR in the marine environment of the study area is seawater.

The mollusks sampled in this study belong to the classes Bivalvia and Gastropoda, which have different feeding behaviors. While bivalves feed by filtering suspended particles, having more direct contact with seawater, limpets and snails are mainly grazing herbivores, scraping food from surfaces (79). However, these differences did not affect the results of this study. When gastropods were excluded from the analysis, the probability of isolating *E. coli* in the mollusk compartment increased slightly, from 0.822 to 0.829. Similarly, the prevalence of florfenicol-NWT *E. coli* in mollusks decreased by only 1%, whereas the prevalence of oxytetracycline, oxolinic acid and flumequine NWT *E. coli* increased by negligible amounts (<0.5%). These changes in probabilities and prevalences did not affect the significance of the statistical models.

Regarding the isolation and resistance patterns observed at the sampling site level, the MCA analysis suggests that in most of the sites, *E. coli* was isolated simultaneously from mollusks and seawater, but not from sediments; only on a few occasions, *E. coli* was found in either seawater or mollusks. This indicates a high degree of concordance in *E. coli* isolation between the seawater and the mollusk compartments. At the same time, the MCA analysis shows that when NWT *E. coli* was detected for florfenicol in seawater, it was generally also detected in the same compartment for oxytetracycline, for oxolinic acid, and for flumequine. Detection of resistant *E. coli* in other compartments was not as consistent among the four antibiotics studied. These results support the idea that seawater is the most appropriate compartment for monitoring AMR of aquaculture origin in the Chilean marine environment.

It is not appropriate to generalize these results beyond the Chilean context because no studies have examined antimicrobial resistance in different environmental compartments simultaneously. Therefore, it is unclear whether the seawater compartment is also the most appropriate for monitoring antimicrobial resistance in other contexts. However, our study’s results reveal patterns that could be replicated elsewhere. For instance, the fact that the seawater compartment was consistently associated with the highest percentage of NWT *E. coli* strains despite the different physicochemical characteristics of the antimicrobials studied, could be due to relatively high levels of fecal contamination (57), together with high emissions of antimicrobials, resistant bacteria, and resistance genes from salmon farms (38, 39), which primarily enter the marine environment through seawater. These conditions are certainly not unique to Chile, so similar results to those of our study could be found in other places with large human settlements and intense aquaculture activity.

## 5 Conclusion

The results of this study confirm that mollusks are suitable organisms for monitoring AMR of aquaculture origin in the marine environment of Chile; however, based on the frequency of isolation of *E. coli* and the frequency of detection of NWT *E. coli*, it appears that monitoring AMR directly from seawater is a more efficient strategy. These results should draw the attention of authorities responsible for designing and implementing antimicrobial resistance action plans in Chile and elsewhere that have decided or are considering using mollusks as environmental sentinels for AMR monitoring, as it is possible that other environmental compartments may be more susceptible to the accumulation of antimicrobial resistance traits.

## Data Availability

The raw data that supports the conclusions of this article can be made available upon request to the corresponding author.
